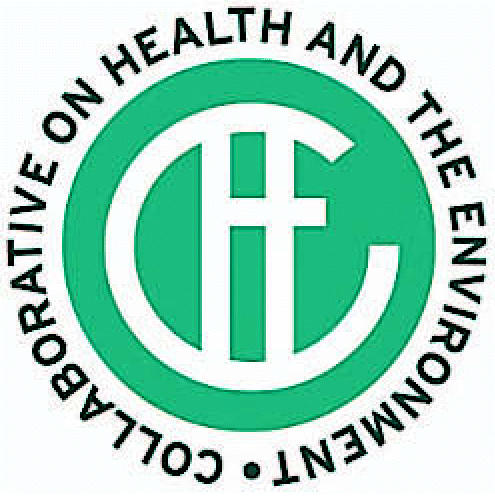# EHPnet: CHE Fertility Online Abstracts Library

**Published:** 2006-11

**Authors:** Erin E. Dooley

A great deal of research is now being published on the effect of environmental chemicals on reproductive health and declines in fertility. The Fertility/Early Pregnancy Compromise Work Group of the Collaborative on Health and the Environment (CHE) is now working to assemble these reports in a searchable abstracts library. This resource was developed by CHE work group member Sarah Janssen along with Pete Myers of EnvironmentalHealthNews.org and Theo Colborn and colleagues at The Endocrine Disruption Exchange. The abstract library is available from a link at **http://www.healthandenvironment.org/wg_fertility_news/652**.

The Bolinas, California–based CHE was formed in the spring of 2002 as a project of the nonprofit health and environmental research institute Commonweal. Its mission is to promote knowledge about the increasing links between human health and environmental toxicants. CHE sponsors a number of work groups that focus on particular areas of concern. EnvironmentalHealthNews.org, meanwhile, is published by the nonprofit organization Environmental Health Sciences, which seeks to help increase public understanding of emerging scientific links between health and the environment. The Endocrine Disruption Exchange seeks to gather, organize, and interpret scientific research relevant to endocrine disruptors.

The listings are updated daily; as of October 2006, the library contained more than 500 news stories, opinion pieces, and scientific studies from sources around the world. The items in the listing are arranged chronologically and have links to the full text of the story when it is available for free.

Links at the side of the abstract listing allow visitors to find items by 1 of 12 options. The first option sorts the entries by article type. Next is a section of 12 Current Issues, which include air, cancer, children’s health, climate change, environmental justice, environmental politics, GMO/bioengineering, hazardous products, reproductive disorders, sewage systems, sustainable business, and water. Following this is a list of 15 human health conditions, contamination agents, exposure pathways, and ecological effects.

Visitors can also search for items sorted by infrastructure (for example, food production or sewage systems), solutions (which encompasses activism, economics, environmental politics, laws, organizing principles, regulations, and sustainable business) and emerging science (including topics such as endocrine disruption and fetal programming). Finally, visitors can find items by area of coverage, publisher, and year of publication, with items dating back as far as 2002.

The library also provides a text search option, which scans all fields or just the title, article text, description, publisher, coverage, or subject. Visitors can add the list as an RSS or JavaScript feed.

As a companion resource, the CHE Fertility/Early Pregnancy Compromise Work Group also has brought together a catalogue of fertility-relevant news stories and organizational reports. This is available from a link on the same page as the abstracts library. Currently, there are more than 1,000 items available within this catalogue, which is set up the same as the abstracts library.

## Figures and Tables

**Figure:**